# Supplementary material for: A NanoBiT assay to monitor membrane proteins trafficking for drug discovery and drug development
Source: Commun Biol. 2022 Mar 8;5:212. doi: 10.1038/s42003-022-03163-9 (PMC8904512; doi:10.1038/s42003-022-03163-9)
Supplement: Supplementary file 2 — Supplementary Information [file 42003_2022_3163_MOESM2_ESM.pdf]

Supplementary Information

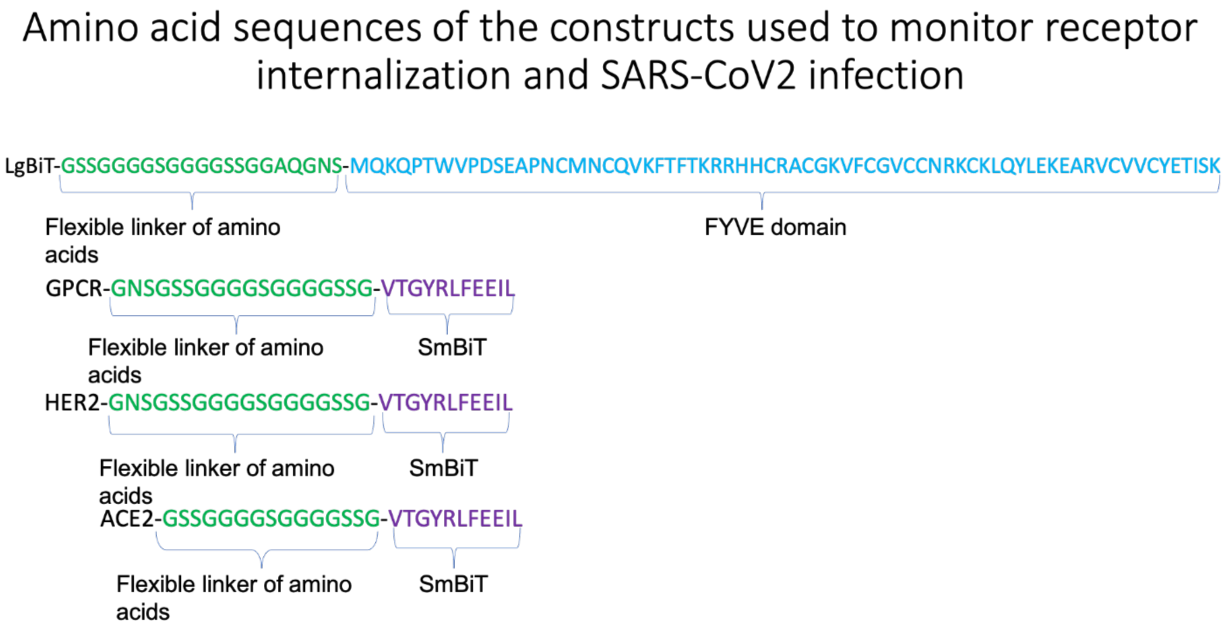

Supplementary Figure-1. Amino acid sequences highlighted in green correspond to the flexible linkers between the receptor and LgBiT or SmBiT. Endofin domain is marked in blue (residues from Q739 to K806).

Amino acid sequences of the constructs used to monitor antibody mediated internalization of FAM19A5 Isoform II

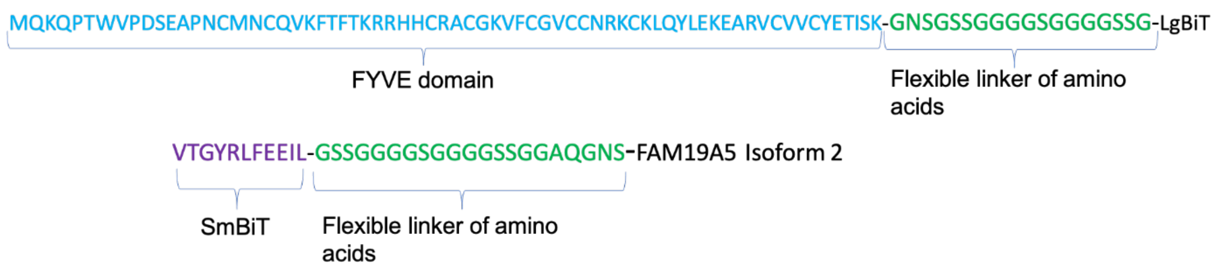

Supplementary Figure-2. Amino acid sequences highlighted in green correspond to the flexible linkers between the receptor and LgBiT or SmBiT. Highlighted sequences in blue correspond to the Endofin domain and in purple to the SmBiT linked to the N-termini of FAM19A5 Isoform II respectively.

Names and DNA sequences of the vectors used to covalently attach LgBiT or SmBiT at the C-termini of the receptors.

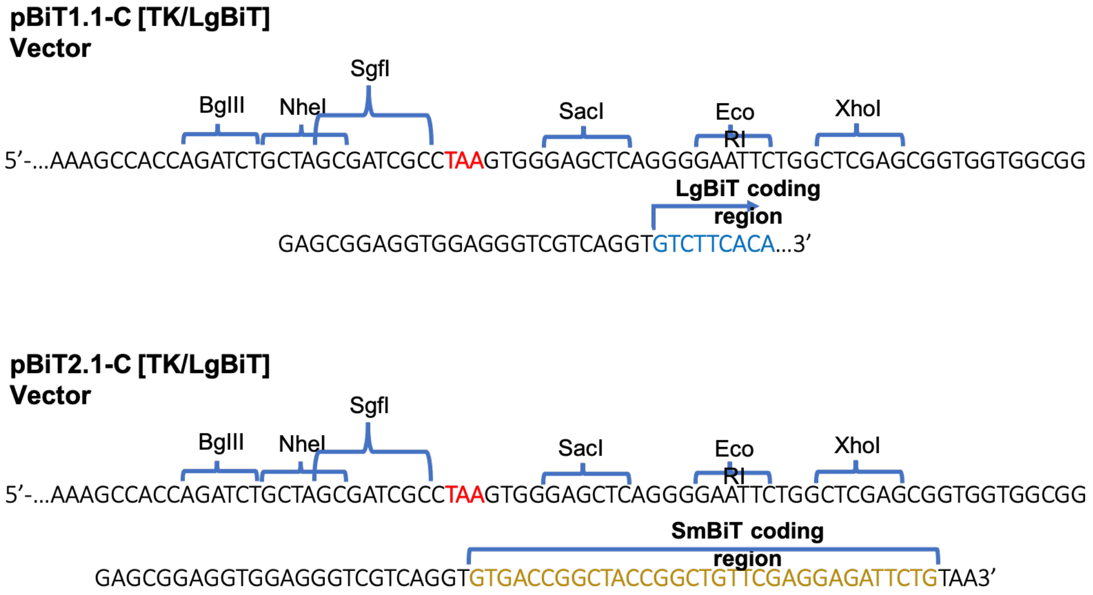

Supplementary Figure 3. Multicloning site sequences showing the corresponding enzyme restriction sites divided by a stop codon (red sequence).

Names and DNA sequences of the vectors used to covalently attach LgBiT or SmBiT at the N-termini of the protein

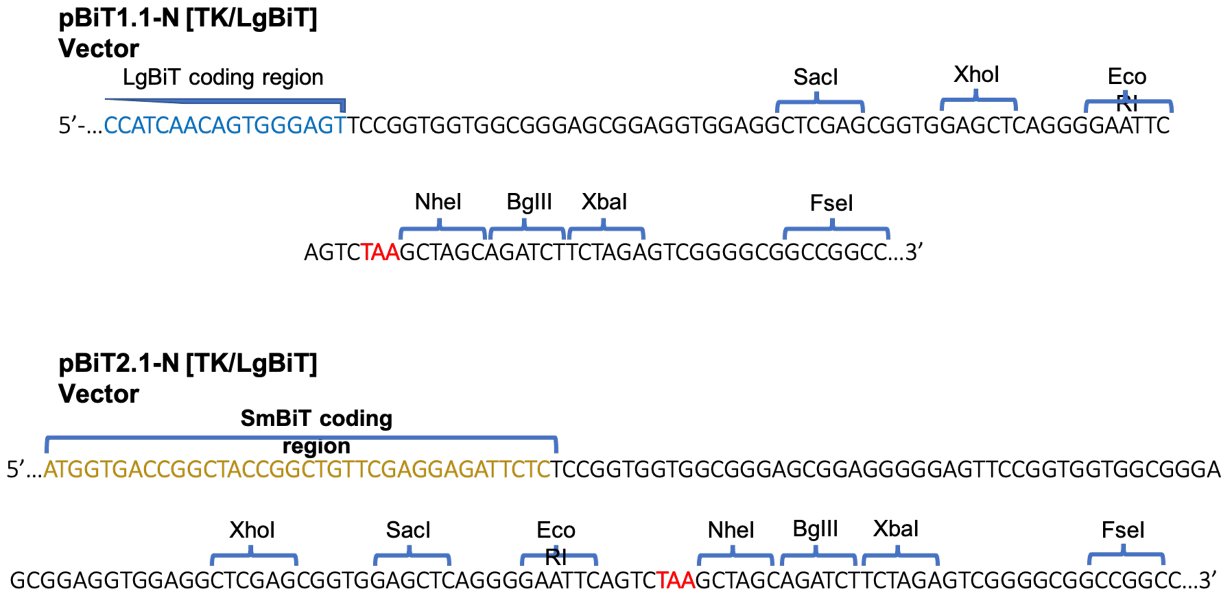

Supplementary Figure-4. Multicloning site sequences showing the corresponding enzyme restriction sites divided by a stop codon (red sequence).

Z-factor as a quality assessment of the NanoBiT internalization assay in different membrane receptor trafficking systems

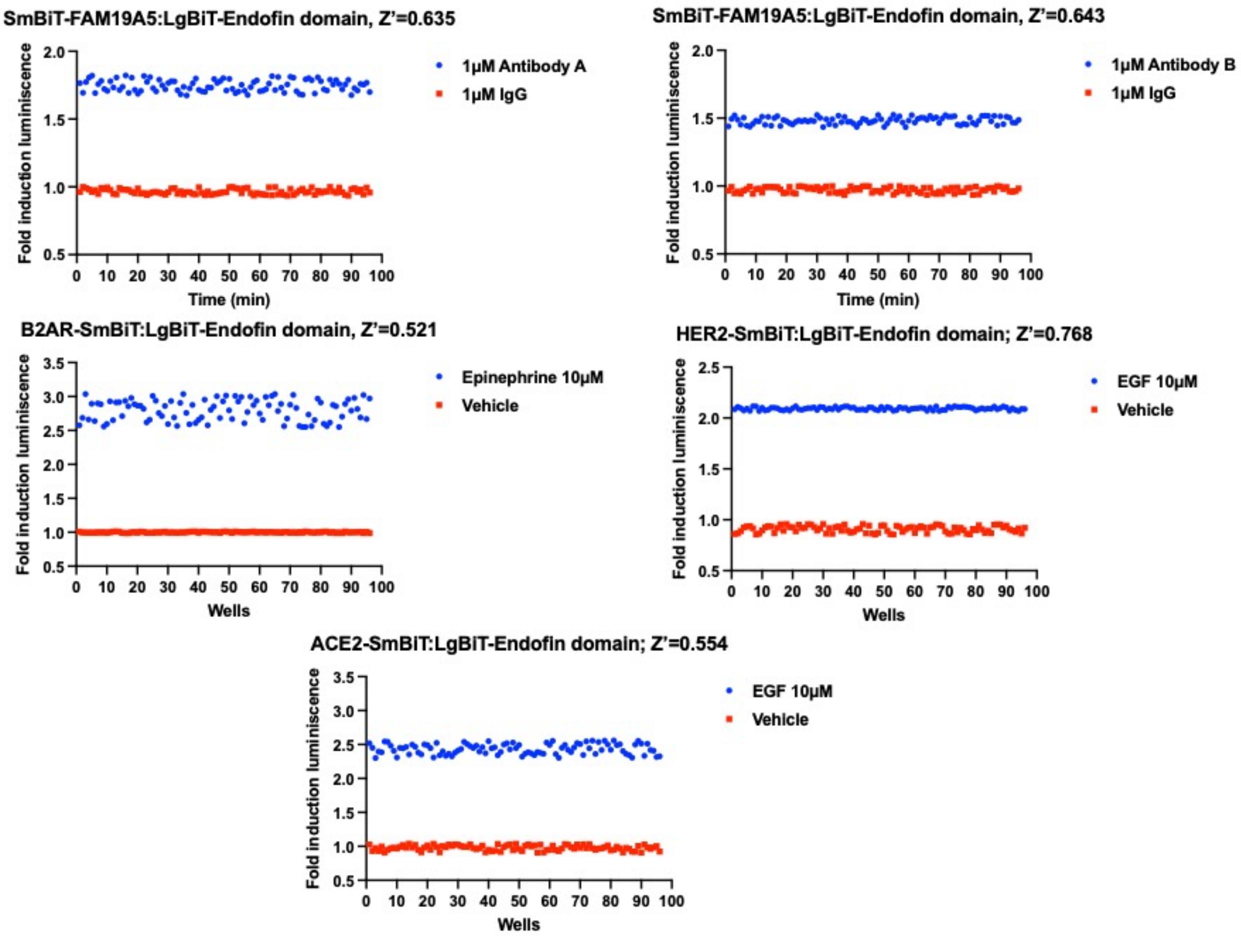

Supplementary Figure-5. Z' factor determination as a quality assessment across different internalization mechanisms.

Histogram taken from bioluminescent imaging at different time points

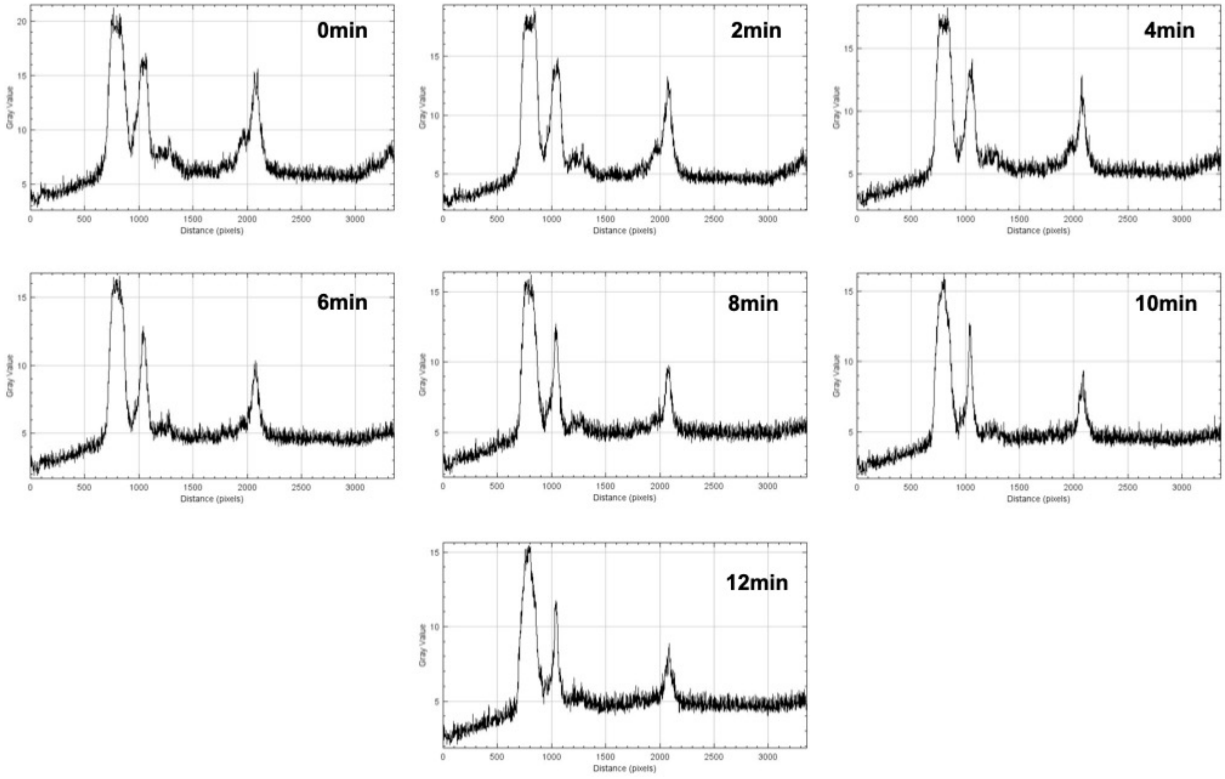

Supplementary Figure-6. Decay in bioluminescence from live cell imaging along the time as a consequence of substrate depletion. A total of 25% substrate depletion was observed within 12 minutes after reaching a maximum in bioluminescence.

Supplementary Table-1. Primer sequences used to clone the corresponding receptor into the internalization NanoBiT system.

Name	Sequence
GAL1 Forward	5’-aagccaccAGATCTACC**ATG**GAGCTGGCGGTCGGG-3’
GAL1 Reverse	5’-CACCACCGCTCGAGCC*CACATGAGTACAATTGGT*-3’
GAL2 Forward	5’-aagccaccAGATCT***GCCGCC***ACCATGAACGTCTCGGGCTGC-3’
GAL2 Reverse	5’-CACCACCGCTCGAGCC*CACATGAGTACAATTGGT*-3’
B2AR Forward	5’-ccagatct*GCTAGC*GCCGCCACCATGGGGCAACCCGGGAAC-3’
B2AR Reverse	5’-TCGAgccaGAATTCCCCAGCAGTGAGTCATTTGT-3’
Endofin Forward	5’-TCGAgccagaattcACAGAAACAGCCTACTTGGGTT-3’
Endofin Reverse	5’-ggtggcttAGATCTTTATTTACTAATAGTTTCATA-3’
FAM19A5 Isoform II Forward	5’-TCGAgccagaattcAATGCAGCTCCTGAAGGCGCTC-3’
FAM19A5 Isoform II reverse	5’-ggtggctt*AGATCT*TCAGGAGACCGTGGTGGTCTTTAT-3’
ACE2 Forward	5’-ccagatct*GCTAGC*GCCGCCACCATGTCAAGCTCTTCCTGGCTCCTT-3’
ACE2 Reverse	5’-CACCACCG**CTCGAG**CTAAAAGGAGGTCTGAACATCATC-3’
Sequencing primer Forward	5-aaggtgacgcgtgtggcctcgaac-3’
Sequencing primer Reverse	5’-gcattttttcactgcattctagtt-3
