# Supplementary material for: A NanoBiT assay to monitor membrane proteins trafficking for drug discovery and drug development
Source: Commun Biol. 2022 Mar 8;5:212. doi: 10.1038/s42003-022-03163-9 (PMC8904512; doi:10.1038/s42003-022-03163-9)
Supplement: Supplementary file 3 — Description of Additional Supplementary Files [file 42003_2022_3163_MOESM3_ESM.pdf]

Description of Additional Supplementary Files

File name: Supplementary Data 1
Description: Underlying data of the graphs in this manuscript.
